# CXCL13 as a Biomarker of Immune Activation During Early and Chronic HIV Infection

**DOI:** 10.3389/fimmu.2019.00289

**Published:** 2019-02-21

**Authors:** Vikram Mehraj, Rayoun Ramendra, Stéphane Isnard, Franck P. Dupuy, Bertrand Lebouché, Cecilia Costiniuk, Réjean Thomas, Jason Szabo, Jean-Guy Baril, Benoit Trottier, Pierre Coté, Roger LeBlanc, Madéleine Durand, Carl Chartrand-Lefebvre, Ido Kema, Yonglong Zhang, Malcolm Finkelman, Cécile Tremblay, Jean-Pierre Routy

**Affiliations:** ^1^Chronic Viral Illness Service, McGill University Health Centre, Montreal, QC, Canada; ^2^Infectious Diseases and Immunity in Global Health Program, Research Institute, McGill University Health Centre, Montreal, QC, Canada; ^3^University of Montreal Hospital Health Centre (CRCHUM), Montreal, QC, Canada; ^4^Department of Microbiology and Immunology, McGill University, Montreal, QC, Canada; ^5^Department of Family Medicine, McGill University, Montreal, QC, Canada; ^6^Clinique Médicale l'Actuel, Montreal, QC, Canada; ^7^Clinique Médicale Quartier Latin, Montreal, QC, Canada; ^8^Clinique Médicale OPUS, Montreal, QC, Canada; ^9^Department of Laboratory Medicine, University Medical Center, University of Groningen, Groningen, Netherlands; ^10^Associates of CapeCod Inc., Falmouth, MA, United States; ^11^Département de Microbiologie, Infectiologie et Immunologie, Université de Montréal, Montreal, QC, Canada; ^12^Hematology Clinic, McGill University Health Centre, Montreal, QC, Canada

**Keywords:** CXCL13, humoral immune response, microbial translocation, inflammation, immune activation, CMV

## Abstract

**Background:** CXCL13 is preferentially secreted by Follicular Helper T cells (T_FH_) to attract B cells to germinal centers. Plasma levels of CXCL13 have been reported to be elevated during chronic HIV-infection, however there is limited data on such elevation during early phases of infection and on the effect of ART. Moreover, the contribution of CXCL13 to disease progression and systemic immune activation have been partially defined. Herein, we assessed the relationship between plasma levels of CXCL13 and systemic immune activation.

**Methods:** Study samples were collected in 114 people living with HIV (PLWH) who were in early (EHI) or chronic (CHI) HIV infection and 35 elite controllers (EC) compared to 17 uninfected controls (UC). A subgroup of 11 EHI who initiated ART and 14 who did not were followed prospectively. Plasma levels of CXCL13 were correlated with CD4 T cell count, CD4/CD8 ratio, plasma viral load (VL), markers of microbial translocation [LPS, sCD14, and (1→3)-β-D-Glucan], markers of B cell activation (total IgG, IgM, IgA, and IgG1-4), and inflammatory/activation markers like IL-6, IL-8, IL-1β, TNF-α, IDO-1 activity, and frequency of CD38^+^HLA-DR^+^ T cells on CD4^+^ and CD8^+^ T cells.

**Results:** Plasma levels of CXCL13 were elevated in EHI (127.9 ± 64.9 pg/mL) and CHI (229.4 ± 28.5 pg/mL) compared to EC (71.3 ± 20.11 pg/mL), and UC (33.4 ± 14.9 pg/mL). Longitudinal analysis demonstrated that CXCL13 remains significantly elevated after 14 months without ART (*p* < 0.001) and was reduced without normalization after 24 months on ART (*p* = 0.002). Correlations were observed with VL, CD4 T cell count, CD4/CD8 ratio, LPS, sCD14, (1→3)-β-D-Glucan, total IgG, TNF-α, Kynurenine/Tryptophan ratio, and frequency of CD38+HLA-DR+ CD4 and CD8 T cells. In addition, CMV+ PLWH presented with higher levels of plasma CXCL13 than CMV- PLWH (*p* = 0.005).

**Conclusion:** Plasma CXCL13 levels increased with HIV disease progression. Early initiation of ART reduces plasma CXCL13 and B cell activation without normalization. CXCL13 represents a novel marker of systemic immune activation during early and chronic HIV infection and may be used to predict the development of non-AIDS events.

## Introduction

Despite the success of antiretroviral therapy (ART), people living with HIV (PLWH) still suffer from chronic immune activation and the development of non-AIDS events (1). Progressive CD4 T cell decline, elevated CD8 T cell counts, early damage to the intestinal mucosa, microbial translocation, CMV co-infection, and persistence of HIV in cellular reservoirs all contribute to systemic immune activation (2–6). Chronic immune activation and inflammation among PLWH on ART have been linked with increased risk for mortality and development of non-AIDS events such as cardiovascular dysfunction, renal failure, hepatic steatosis, neurocognitive degeneration, accelerated aging, and cancer (7, 8). Therefore, therapeutic strategies are needed to tackle residual immune activation to improve the quality of life of PLWH.

Apart from the virus itself or its proteins, factors contributing to immune activation among PLWH include elevated circulation of microbial products leaking from the gut and CMV infection (9, 10). It has been reported that epithelial gut damage, leading to microbial translocation, precedes systemic immune activation in a SIV-infected rhesus macaque model of the disease (11). Circulation of gram-negative bacterial cell wall antigen, lipopolysaccharide (LPS), and major component of fungal cell walls, (1→3)-β-D-Glucan (βDG), have been reported to be elevated in the plasma of PLWH (12–16). These markers of microbial translocation have been previously associated with activated CD4 and CD8 T cells, indoleamine 2,3-dioxygenase 1 (IDO-1) activity, and plasma levels of inflammatory cytokines. Thus, the optimal marker of systemic immune activation in PLWH should account for the contribution of circulating microbial products LPS and βDG.

Several cellular and soluble markers of immune activation/inflammation have been identified (17). Frequencies of HLA-DR and CD38 expressing CD4 and CD8 T cells are used to measure lymphoid cell activation (18). Inflammatory myeloid cells including monocytes, macrophages, and dendritic cells (DC) are reported to induce IDO-1 enzyme activity upon antigen stimulation (19). IDO-1 converts essential amino acid tryptophan into immune suppressive kynurenines. Frequency of activated CD4 and CD8 T cells as well as IDO-1 activity of myeloid cells have been associated with the degree of epithelial gut damage, microbial translocation, and size of HIV reservoir (1, 20–22). Plasma level markers of inflammation/immune activation include marker of monocyte activation soluble CD14 (sCD14) as well as inflammatory cytokines such as IL-6, IL-1β, IL-8, and TNF-α which are secreted by activated immune cells (23–25). While there are several validated markers of immune activation/inflammation in PLWH, there is currently no single marker that can account for both lymphoid and myeloid cell activation during early and chronic stages of HIV infection.

B cells play a crucial role in mounting an antiviral humoral immune response. Upon maturation in germinal centers (GCs), they are mainly localized in secondary lymphoid tissues where they present antigens, secrete cytokines, and produce antigen-specific antibodies (26). PLWH have been reported to have humoral immune dysfunction that is regulated by GCs, which have been linked to HIV-associated immune activation (27–29). Both structural and functional impairment of lymph node GCs lead to B cell dysfunction. Subsequent B cell activation results in hypergammaglobulinemia, impaired antibody response, and loss of B cell memory (27). B cell activation and trafficking to lymph node GCs is controlled by secretion of chemokine CXCL13 [(C-X-C motif) ligand 13] that is recognized by leukocytes, which express CXCR5. As such, CXCL13 is considered as a marker of GC activity as it is secreted by follicular DC and helper T (T_FH_) cells. Furthermore, myeloid cells such as macrophages have also been reported to secrete CXCL13 upon activation (30). It has been previously reported that PLWH have elevated plasma levels of CXCL13 during chronic infection (31). However, plasma levels of CXCL13 during early infection, the effect of ART on CXCL13 secretion, and the contribution of CXCL13 to HIV disease progression and systemic immune activation are poorly understood. Herein, we assessed the validity of plasma levels of CXCL13 as a biomarker of systemic immune activation in a well-defined cohort of early and chronic, untreated and treated PLWH.

## Methods

### Study Design and Population

114 adult PLWH were cross-sectionally grouped into those in early HIV-infection (EHI) (*n* = 37), defined as being within 6 months of the estimated date of infection, and those with chronic HIV-infection (CHI) who were either untreated (*n* = 13) or ART treated (*n* = 64). EHI participants were enrolled from the Montreal Primary HIV Infection Study (32); while CHI participants were enrolled from the Chronic Viral Illness Service at the McGill University Health Centre and Canadian HIV and Aging Cohort Study (33). In addition, 35 elite controllers (EC)s, defined as PLWH who control plasma viral loads below 50 copies per mL and maintain CD4 T-cell counts above 500 cells per mm^3^ in the absence of ART were included from the Canadian Cohort of HIV-infected Slow Progressors (34). Within the EHI group, 24 participants were prospectively followed-up for about 2 years. During the follow-up, 10 EHI participants were on ART for at least 1 year while the remaining participants were ART naïve during the time of longitudinal assessment. A group of 17 HIV-uninfected controls (UC) were assessed for comparison with EHI and CHI groups.

### Laboratory Measurements

Participants were diagnosed with HIV by measuring plasma HIV-1 p24 antigen/antibody and were further confirmed by Western blot as previously reported (32, 35). HIV viral load (VL) in plasma was quantified by the Abbott RealTime HIV-1 assay (Abbott Laboratories, Abbott Park, Illinois, U.S.A). Assessment of CD4 and CD8 T cell counts was done by 4-color flow cytometry. For further research measurements blood samples of study participants were collected to isolate plasma and peripheral blood mononuclear cells (PBMC) samples and stored at −80°C and in liquid nitrogen, respectively. All participants were fasting at the time of blood collection.

### Quantification of Plasma Levels of CXCL13

Plasma CXCL13 levels were measured in duplicate by using the Human CXCL13/BLC/BCA-1 Quantikine ELISA Kit (R&D Systems, Minneapolis, MN), a 4.5-h solid phase enzyme linked immunosorbent assay (ELISA).

### Quantification of Markers of B-Cell Activity (Total IgG, IgM, IgA, IgG1-4, BAFF, sCD40L)

Total IgG, IgM, and IgA were measured using the Olympus AU5800 (Beckman Coulter). Further subclasses of IgG (IgG1, IgG2, IgG3, and IgG4) were measured by using ELISA kits (eBiosciences, Saint Laurent, QC, Canada) as per manufacturer's instructions. B cell activating factor (BAFF) and soluble CD40L (sCD40L) were measured in duplicate using an ELISA (R&D Systems, Minneapolis, MN, USA).

### Quantification of Markers of Epithelial Gut Damage and Microbial Translocation

Intestinal-fatty acid binding protein (I-FABP) was measured using an ELISA kit (Hycult Biotech, Uden, Netherlands). Soluble suppressor of tumorigenicity 2 (sST2) was measured by ELISA as described before (21). LPS was measured using a human lipopolysaccharide ELISA kit (Cusabio, Wuhan, China). sCD14 was measured by immunoassay (Quantikine, R&D Systems, Minneapolis, MN, USA). (1→3)-β-D-Glucan (βDG) was measured by the Fungitell® Limulus Amebocyte Lysate assay (Associates of Cape Cod, Inc., East Falmouth, MA, USA). All the analytes were measured in duplicate as per manufacturer's instructions.

### Multiplex Quantification of Soluble Inflammatory Markers

Plasma levels of IL-1β, Tumor Necrosis Factor α (TNF-α), IL-6, and IL-8 were measured in duplicate using the Meso Scale Discovery (MSD) U-Plex Pro-Inflammatory Combo 4 kit (MSD, Rockville, Maryland, USA).

### Measurement of Kynurenine and Tryptophan Plasma Levels

Kynurenine and Tryptophan were measured using an automated on-line solid-phase extraction-liquid chromatographic-tandem mass spectrometric method (36, 37). Ratio of kynurenine to tryptophan was calculated as a measure of IDO-1 enzyme activity.

### Flow Cytometry Analyses

Frozen PBMC samples were rapidly thawed and stained for 20 min at 4°C using fluorochrome conjugated antibody panels from BD Biosciences (Mississauga, ON, Canada) or BioLegend (San Diego, CA, USA) using anti-CD56-FITC, anti-CD11c BV711, anti-CD3 PE-Dazzle594, anti-CD4 BUV395, anti-CD8 BUV737, anti-CD38 BV605, anti-HLADR APC-Cy7. Cells were then washed and fixed in 2% paraformaldehyde before acquisition. CD38 and HLA-DR expression were analyzed on CD4 and CD8 T cells. Dead cells were excluded as Live/dead positive (ThermoFisher, Saint Laurent QC, Canada). Fluorescence minus one color controls were used to discriminate auto-fluorescence from positive signals. BD Fortessa X20 flow cytometer was used for FACS and the data was analyzed using FlowJo 10.0.7 (FLOWJO, LLC, Ashland, OR, USA).

### *In vitro* Stimulations

One million PBMC from uninfected donors were stimulated for 18 h in 1 mL complete medium [RPMI (Wisent) + 10% FBS (Wisent) + 1% Penicillin/Streptomycin (ThermoFisher)] with 1 μg/ml of LPS (Sigma), βDG (Sigma) or both. Supernatants were collected and CXCL13 was measured as previously described.

### Statistical Analyses

Descriptive and inferential analyses were conducted using SPSS 24.0 (Chicago, IL, USA) and GraphPad Prism 6.0 (La Jolla, CA, USA). Data were summarized using means and standard deviations calculated for the variables with normal distribution and using median with interquartile range (IQR) calculated for variables with a non-normal distribution. Percentages were calculated for categorical variables. Subsequently, both parametric and non-parametric tests including student t, chi-square, Mann-Whitney *U*, χ^2^, and ANOVA with LSD test were used for comparisons. Paired *t*-test was used for before-after comparisons. Pearson correlation test was conducted to assess association between two quantitative variables. Statistical significance was determined at *p* < 0.05. Multivariate linear regression analysis was conducted to determine the independent association of CXCL13 with HIV-infection adjusting for the confounding factors such as age, sex, CD4 T cell count and inflammatory markers.

## Results

### Clinical Characteristics of Study Participants

Eighty one percent of the participating PLWH were male and the median (IQR) age was 47 (37–51). CD4 T-cell count was lower in untreated EHI and CHI vs. controls, which increased amongst those receiving antiretroviral therapy (ART). In contrast, CD8 T-cell count among untreated PLWH was higher than those receiving ART. Untreated EHI and CHI groups had a median log_10_ plasma viral loads of 4.4 (3.7–5.0) and 5.1 (4.6–5.3) copies per mL, respectively. All the participants on ART and all ECs had plasma viral loads <50 copies/mL (Table 1).

**Table 1:** Descriptive characteristics of study participants (*n* = 166).

**Characteristics**	**EHI (*n* = 37)**	**CHI ART– (*n* = 13)**	**CHI ART+ (*n* = 64)**	**EC (*n* = 35)**	**UC (*n* = 17)**
**Age in years**					
Median (IQR)	34 (28–44)	43 (33–51)	50 (55–61)	45 (39–50)	41 (35–49)
**Sex**					
Male, *n* (%) Female, *n* (%)	36 (97.3) 1 (2.7)	8 (38.5) 5 (61.5)	58 (90.6) 6 (9.4)	21 (60.0) 14 (40.0)	11 (64.7) 6 (35.3)
**CD4 T cells/μL**					
Median (IQR)	480 (368–650)	81 (14–319)	605 (412–700)	650 (552–823)	866 (566–1,022)
**CD8 T cells/μL**					
Median (IQR)	810 (630–1,030)	1,030 (267–1,232)	746 (554–1,001)	690 (409–968)	408 (281–689)
**CD4/CD8**					
Median (IQR)	0.6 (0.4–0.9)	0.1 (0.1–0.4)	0.8 (0.5–1.1)	1.1 (0.8–1.4)	1.7 (1.2–3.0)
**VL (log**_**10**_ **copies/mL)**					
Median (IQR)	4.4 (3.7–5.0)	5.1 (4.6–5.3)	< 1.7	< 1.7	NA

*EHI, early HIV infection; ART, antiretroviral therapy; CHI, chronic HIV infection; EC, elite controllers; UC, uninfected controls; NA, not applicable*.

### Elevation of Plasma CXCL13 Levels During Early and Chronic HIV Infection and Impact of Antiretroviral Therapy

**Figure 1:**
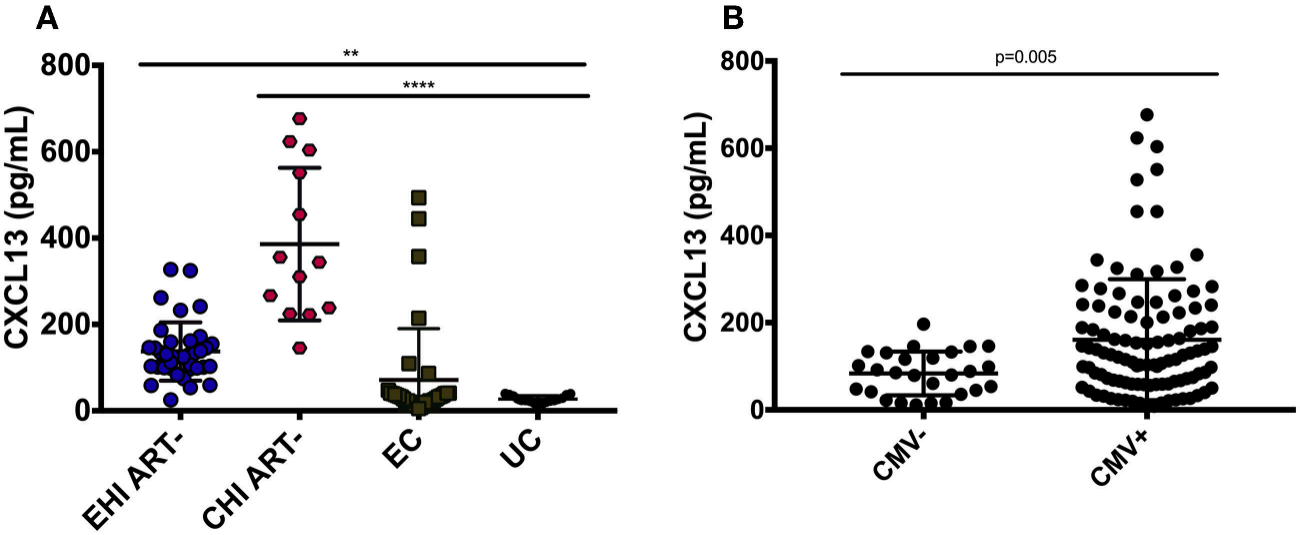
Plasma levels of CXCL13 over the course of HIV-infection. **(A)** Circulating CXCL13 during early and chronic infection compared to elite controllers and uninfected controls. EHI ART– (*n* = 37), CHI ART– (*n* = 13), EC (*n* = 35), UC (*n* = 17). **(B)** Comparison of plasma levels of CXCL13 in CMV+ and CMV– HIV-infected progressors with similar CD4 T cell count. EHI, early HIV infection; CHI, chronic HIV infection; EC, elite controllers; UC, uninfected controls; ART, antiretroviral therapy. **p < 0.01; ****p < 0.0001.

**Figure 2:**
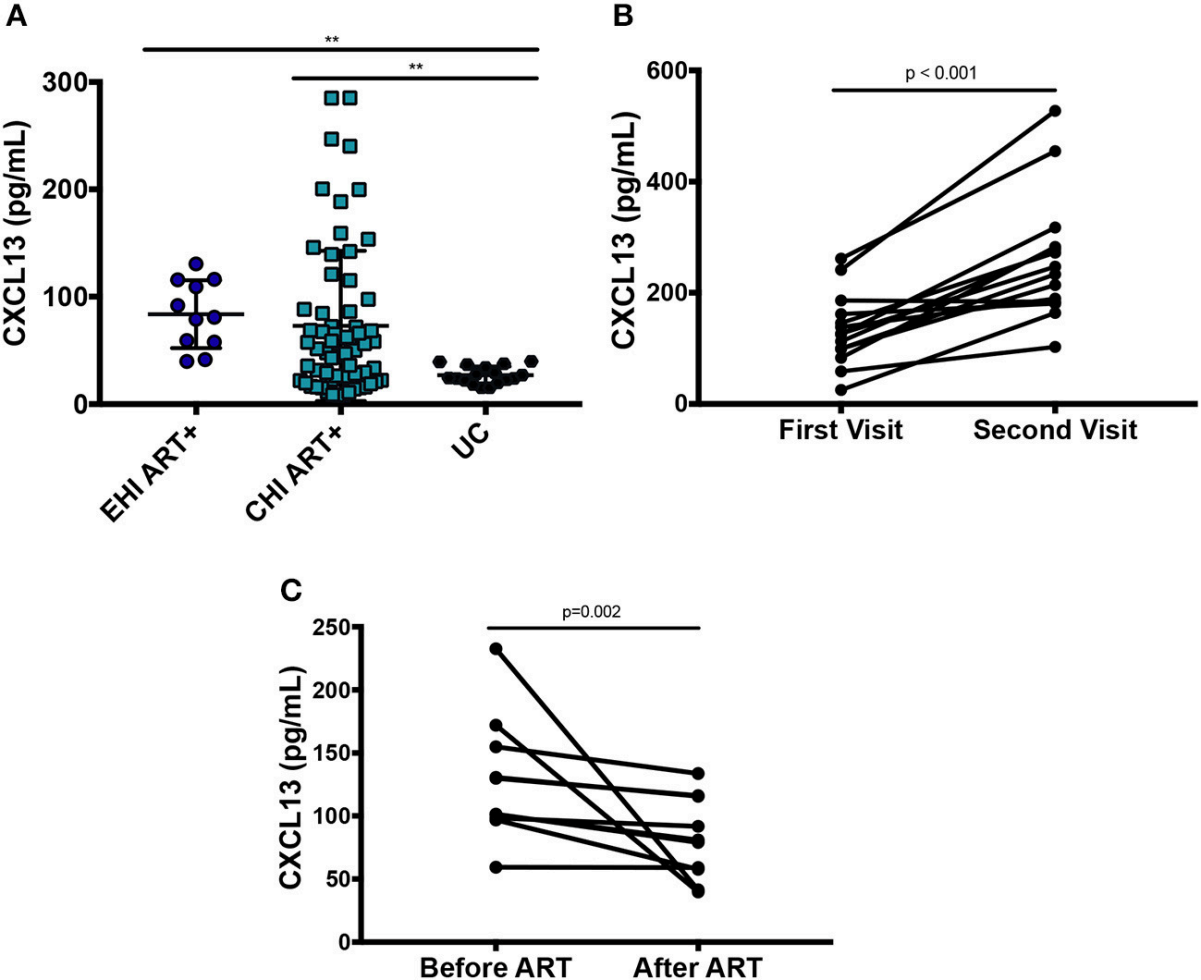
Effect of ART on plasma levels of CXCL13. **(A)** Cross-sectional analysis on the effect of ART on plasma levels of CXCL13 (EHI ART+ *n* = 11; CHI ART+ *n* = 64; UC *n* = 17). **(B)** Longitudinal analysis on the change of plasma levels of CXCL13 over 24 months in PLWH without ART (*n* = 14). **(C)** Longitudinal analysis on the change of plasma levels of CXCL13 in PLWH after 24 months on ART (*n* = 10). EHI, early HIV infection; CHI, chronic HIV infection; UC, uninfected controls; ART, antiretroviral therapy. **p < 0.01.

Plasma levels of CXCL13 were significantly elevated in ART-naive EHI (137.3 ± 67.4 pg/mL) and ART-naive CHI (385.91 ± 76.8 pg/mL) compared to EC (71.3119.0 pg/mL) and UC (33.414.9 pg/mL) (Figure 1A). EC had lower levels of plasma CXCL13 than EHI and CHI but still had nearly two-fold more CXCL13 than UC. However, this difference was not statistically significant. This is likely because EC are known to have a comparatively preserved immune system compared to progressors (38). We observed PLWH with CMV co-infection to have higher plasma levels of CXCL13 than PLWH without CMV co-infection (*p* = 0.005) (Figure 1B). Of note, CMV co-infection has been reported to be linked to HIV-associated immune activation (39). In cross-sectional analysis, both EHI and CHI groups on ART compared to their untreated counterparts showed a significant decrease in their CXCL13 levels without normalization. Longitudinal analysis of the EHI participants that remained without ART for a median of 24 months demonstrated that their plasma CXCL13 levels increased from 133.2 ± 17.3 to 260.5 ± 30.4 pg/mL (*p* < 0.001). On the other hand, EHI participants who received ART for a median of 24 months showed a decrease in their CXCL13 levels without normalization (81.6 ± 10.3 pg/mL, *p* = 0.002) (Figure 2). It is important to note that while there is an overall trend of reduced plasma levels of CXCL13 after 24 months of ART, such reduction is pronounced in two participants.

These results suggest that plasma levels of CXCL13 progressively increases with HIV disease progression and is decreased without normalization upon initiation of ART. This is in line with previous findings that ART results in a decrease without normalization of systemic immune activation (40). Multivariate analysis confirmed that elevated CXCL13 levels among PLWH were independent of the effect of age, sex, HIV viral load, CD4 T cell count, and inflammatory markers.

### Correlation of Plasma CXCL13 Levels With CD4 and CD8 T-Cell Counts, CD4/CD8 T Cell Ratio and Plasma Viral Load

**Figure 3:**
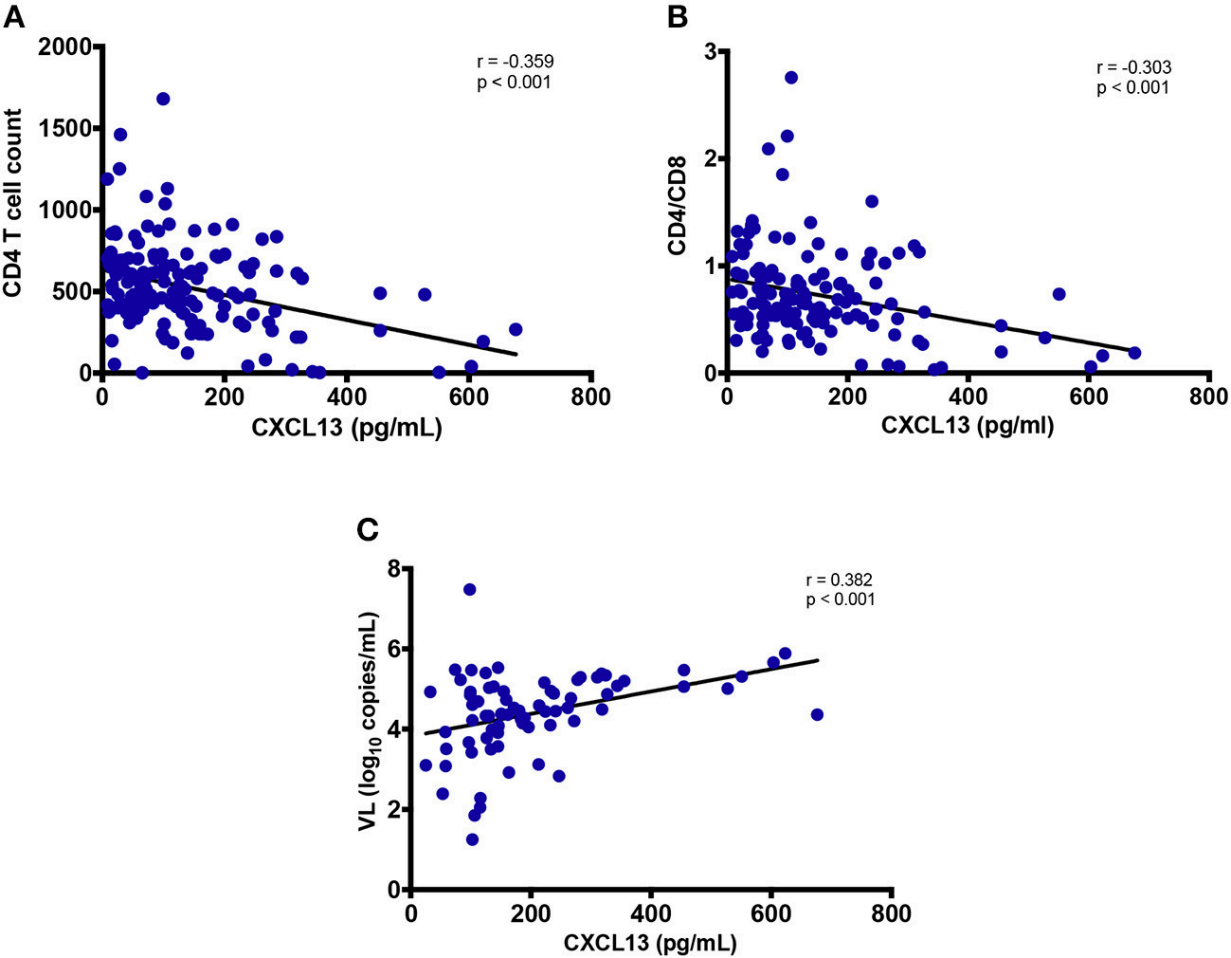
Association of plasma levels of CXCL13 with markers of HIV disease progression. **(A)** Plasma levels of CXCL13 are correlated with CD4 T cell count in HIV-infected progressors (*n* = 144). **(B)** Plasma levels of CXCL13 are correlated with CD4/CD8 T cell ratio in HIV-infected progressors (*n* = 144). **(C)** Plasma levels of CXCL13 are correlated with plasma viral load in untreated PLWH (*n* = 72).

Among PLWH, CXCL13 levels correlated negatively with both CD4 T-cell count (*r* = −0.359; *p* < 0.001) and CD4/CD8 T cell ratio (*r* = −0.303; *p* < 0.001) but not with CD8 T-cell count (*r* = 0.107; *p* = 0.232, data not shown). Elevated CXCL13 levels among untreated participants (both EHI and CHI) positively correlated with viral load (*r* = 0.382; *p* < 0.001) (Figures 3A–C) (41).

### Correlation of Plasma CXCL13 Levels With B-Cell Activation Markers

**Figure 4:**
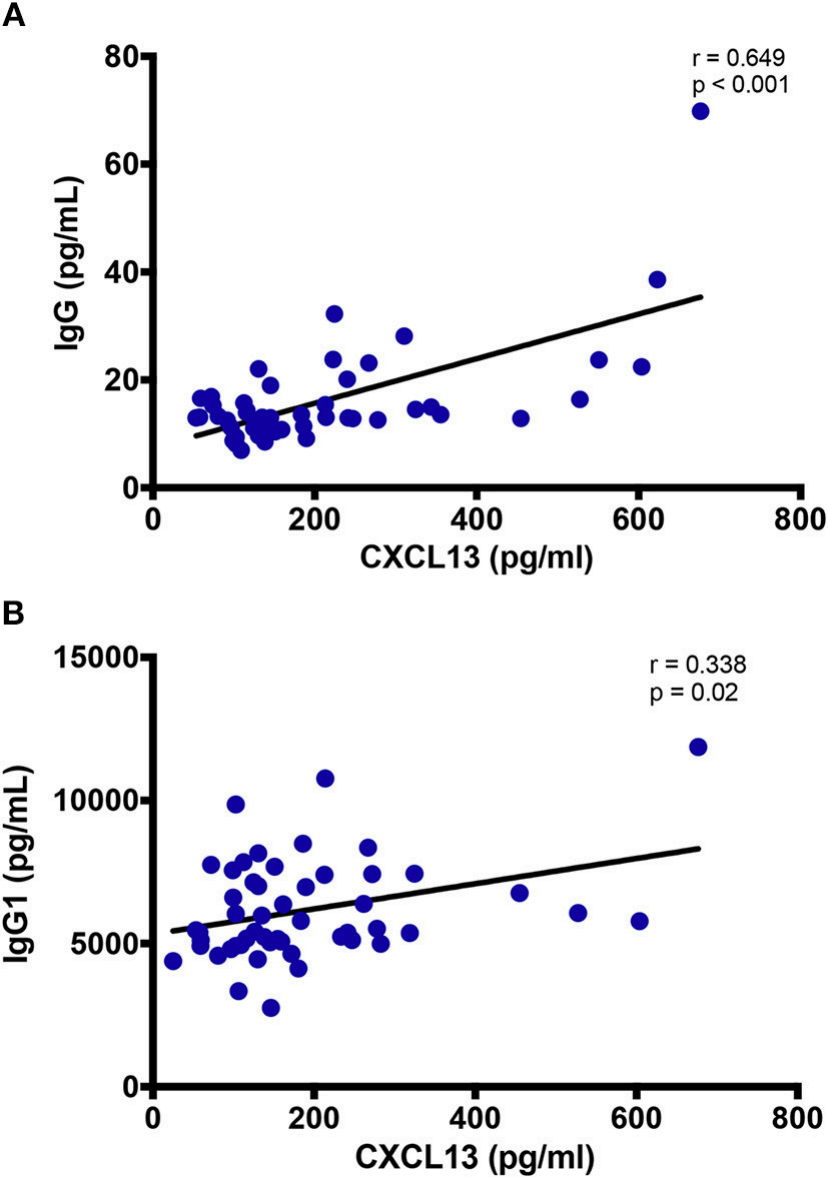
Plasma levels of CXCL13 are associated with immunoglobulin production. **(A)** Plasma levels of CXCL13 are correlated with plasma levels of non-specific IgG in a subset of EHI and CHI PLWH, including participants on and off ART (*n* = 49). **(B)** Plasma levels of CXCL13 are correlated with plasma levels of non-specific IgG1 in a subset of EHI and CHI PLWH, including participants on and off ART (*n* = 51).

CXCL13 levels correlated with total IgG (*r* = 0.649; *p* < 0.001), and IgG1 (*r* = 0.338; *p* = 0.02) in circulation (Figures 4A,B), while no correlation was observed with IgG2, IgG3, nor IgG4 (data not shown). Similarly, despite a positive trend, the correlation of plasma CXCL13 was not significant with plasma BAFF and sCD40L levels, two other indicators of B-cell activity (data not shown). These results highlight the distinctive contribution of CXCL13 in B-cell activation and humoral immune response.

### Correlation of Plasma CXCL13 Levels With Soluble Markers of Gut Damage and/or Microbial Translocation

**Figure 5:**
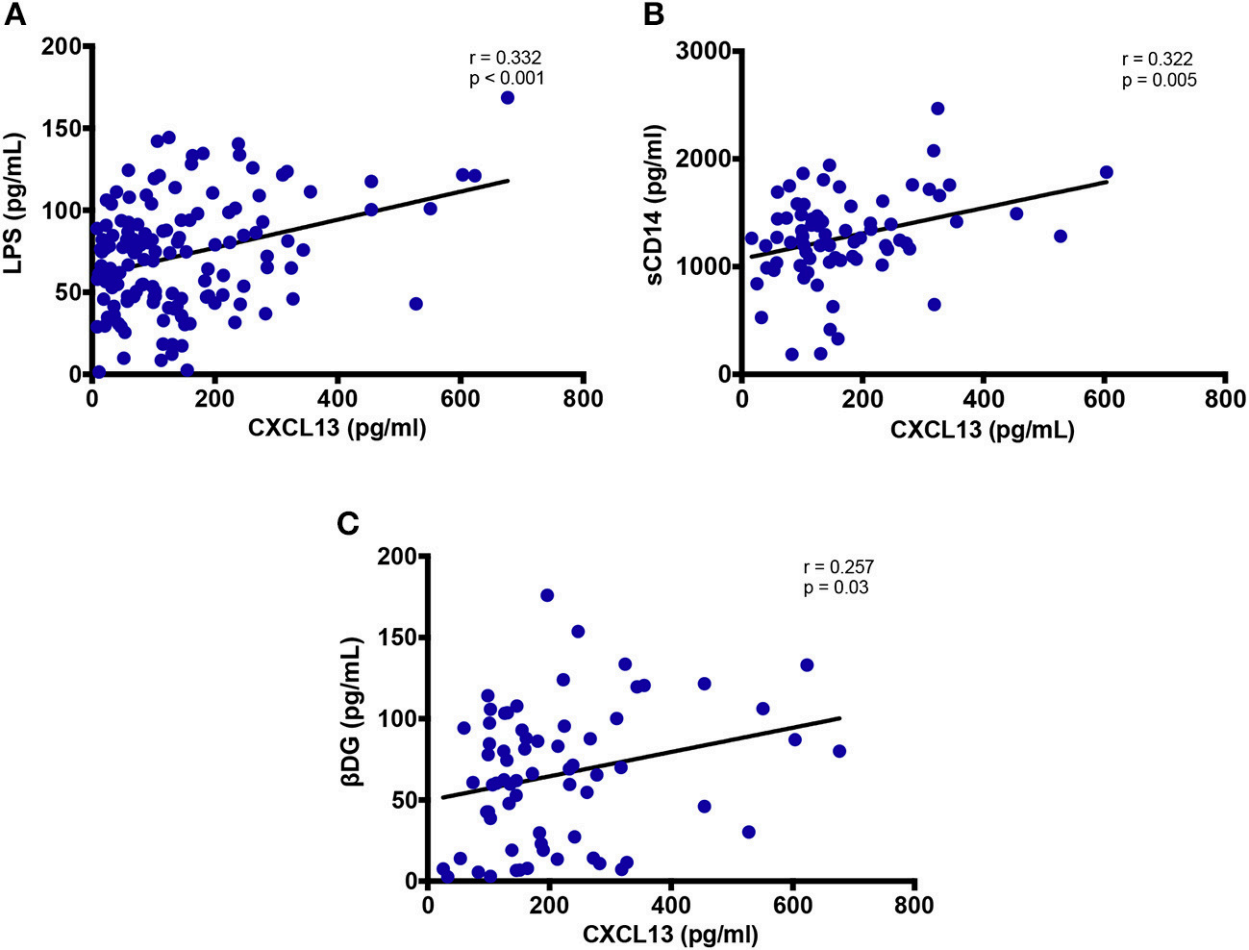
Plasma levels of CXCL13 are associated with markers of microbial translocation. **(A)** Plasma levels of CXCL13 are correlated with plasma levels of LPS (*n* = 144). **(B)** Plasma levels of CXCL13 are correlated with plasma levels of soluble CD14 (sCD14), in a subset of EHI and CHI PLWH, including those on and off ART (*n* = 74). **(C)** Plasma levels of CXCL13 are correlated with plasma levels of (1→3)-β-D-Glucan (βDG), in a subset of EHI and CHI PLWH, including those on and off ART (*n* = 68).

Plasma CXCL13 did not correlate with marker of epithelial gut damage, I-FABP (data not shown). We also assessed sST2 as an emerging marker of gut damage (21). CXCL13 levels did not show any correlation with sST2 (data not shown). However, plasma levels of gram-negative bacterial cell wall endotoxin LPS and monocyte activation marker sCD14 positively correlated with systemic CXCL13 levels (*r* = 0.332; *p* < 0.001 and *r* = 0.322; *p* = 0.005, respectively) (Figures 5A,B). Both LPS and sCD14 are considered as markers of bacterial translocation. In addition, we also measured fungal translocation using a major fungal cell wall polysaccharide antigen, βDG (42). Plasma levels of βDG correlated significantly with plasma CXCL13 (*r* = 0.257; *p* = 0.03) (Figure 5C). Of note, βDG has been used as a diagnostic marker for invasive fungal infections and its role is emerging in HIV-associated fungal translocation as we and others have previously reported (13, 15, 43). *In vitro*, both LPS and βDG induced CXCL13 secretion by PBMC from uninfected donors. Combination of both LPS and βDG did not show an additive effect on CXCL13 secretion compared to LPS or βDG alone (data not shown).

### Correlation of Plasma CXCL13 Levels With Soluble Markers of Inflammation and/or Immune Activation

**Figure 6:**
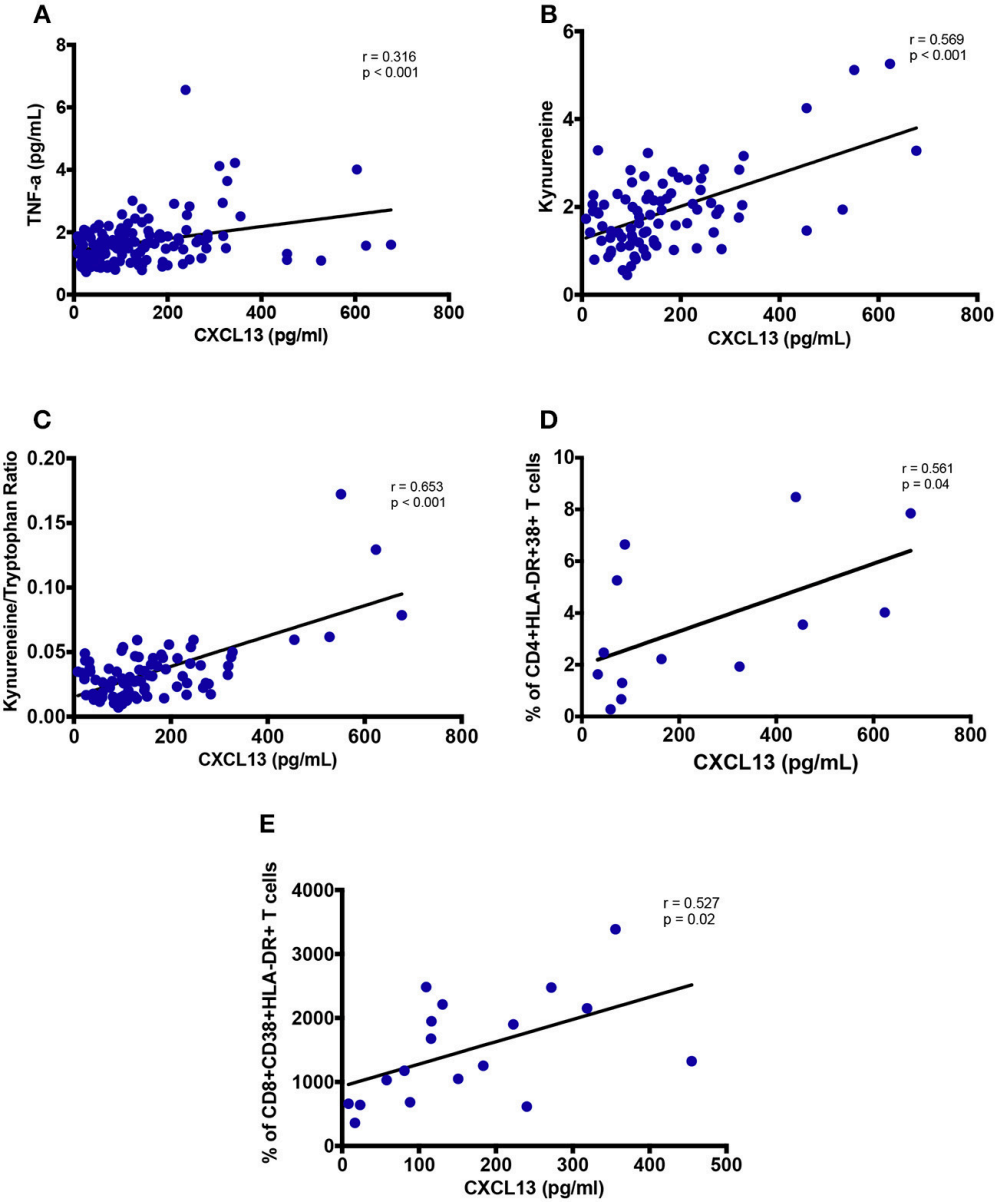
Plasma levels of CXCL13 are associated with markers of myeloid and lymphoid activation. **(A)** Plasma levels of CXCL13 are correlated with plasma levels of TNF-α (*n* = 139). **(B)** Plasma levels of CXCL13 are correlated with plasma levels of Kynurenine, in a subset of PLWH in early and chronic stages of infection, including those on and off ART (*n* = 84). **(C)** Plasma levels of CXCL13 are correlated with IDO-1 metabolism (Kynurenine/Tryptophan), in a subset of EHI and CHI participants including those on and off ART (*n* = 84). **(D)** Plasma levels of CXCL13 are correlated with frequency of HLA-DR^+^CD38^+^ CD4 T cells, in a random subset of PLWH from early and chronic HIV-infection (*n* = 13). **(E)** Plasma levels of CXCL13 are correlated with frequency of HLA-DR+CD38+ CD8 T cells, in a random subset of PLWH from early and chronic HIV-infection (*n* = 18).

Plasma TNF-α levels were observed to have a significant positive correlation with circulating CXCL13 levels (*r* = 0.316; *p* < 0.001, Figure 6A), while no correlation of CXCL13 was observed with other inflammatory markers IL-1β, IL-6, and IL-8 (data not shown). We also quantified plasma Tryptophan and Kynurenine levels to measure IDO-1 enzyme activity as a marker of myeloid cell inflammation and/or immune activation. Plasma CXCL13 levels had a significant positive correlation with plasma kynurenine (*r* = 0.569; *p* < 0.001, Figure 6B). We computed ratio of kynurenine to tryptophan as a measure of IDO-1 enzyme activity. This ratio further significantly correlated (*r* = 0.653; *p* < 0.001, Figure 6C) with plasma CXCL13 levels. Taken together, these results portray the involvement of CXCL13 in HIV-associated myeloid cell activation and inflammation.

### Correlation of Plasma CXCL13 Levels With Lymphocyte Activation Markers

We analyzed PBMC by FACS for whom samples were available. Frequency of CD38^+^HLA-DR^+^ CD4 T cells (*r* = 0.561; *p* = 0.04, Figure 6D) and frequency of CD38^+^HLA-DR^+^ CD8 T cells (*r* = 0.527; *p* = 0.02, Figure 6E) correlated with plasma levels of CXCL13. These findings highlight CXCL13 as a plasma level biomarker of lymphoid cell activation.

## Discussion

We report significant elevation of plasma CXCL13 levels during early and chronic HIV infection that reduced without normalization in PLWH receiving antiretroviral therapy. To our knowledge, this is the first observation reporting a correlation of CXCL13 levels with markers of microbial translocation, inflammatory cytokines, IDO-1 enzyme activity and markers of myeloid and lymphoid cell activation in PLWH. In addition, we also showed a significant correlation of plasma CXCL13 levels with markers of B cell activation in the context of HIV infection. Interestingly, plasma levels of CXCL13 were also associated with CMV co-infection which was reported to be a contributor to HIV-associated systemic inflammation (39). Immune activation is considered as a major factor contributing to the size of HIV reservoirs and development of non-AIDS events despite long-term ART (1, 4, 7). Therefore, therapeutic strategies targeting immune activation remains a priority for curing HIV infection.

Our results are in line with the first report of elevated CXCL13 levels in PLWH. Widney et al. observed a positive correlation of plasma CXCL13 with inflammatory markers and with CD4 T cell count in chronically infected patients (31). In contrast, we observed a negative correlation of CXCL13 with CD4 T cell counts and CD4/CD8 T cell ratio. Progressive decline in the CD4 T cell numbers and CD4/CD8 T cell ratio is a hallmark of HIV-infection (44, 45). Therefore, their negative correlation with CXCL13 levels explains a role of the later in HIV-disease progression via immune activation. Furthermore, in untreated participants, we observed a correlation of plasma CXCL13 with viral load. In untreated participants, the associations of plasma CXCL13 with CD4 T cell count, CD4/CD8 ratio, LPS, sCD14, βDG, and IDO-1 metabolism were independent of HIV viral load.

Moreover, Widney et al. did not observe an association of CXCL13 levels and immunoglobulins. On the other hand, we found a correlation of CXCL13 with IgG and more specifically with its predominant subclass IgG1. This observation shows concordance of plasma CXCL13 levels with B cell activation, which is mediated via CXCR5 expression (46).

Chronic immune activation and inflammation are understood to be the driving factors of HIV disease progression and a significant underlying cause of non-AIDS events in PLWH on long-term ART. As such, it is of clinical interest to validate a biomarker that can monitor myeloid and lymphoid cell activation/inflammation in early and chronic stages of infection in both progressors and non-progressors. In these lines, T_FH_ cells are reported to secrete CXCL13 (47). Cohen et al. have shown that HIV-1 ssRNA induces the production of CXCL13 by DC (48). Furthermore, Carlsen et al. showed that LPS stimulates macrophages to produce CXCL13 *in vitro* (30). We show plasma levels of CXCL13, measured by ELISA, to be a valid marker of HIV disease progression (CD4 T cell count and plasma viral load), microbial translocation (circulation of LPS and βDG), myeloid cell activation (IDO-1 activity and plasma sCD14 levels), lymphoid cell activation (frequency of CD38+ HLA-DR+ CD4 and CD8 T cells), and general inflammation (plasma levels of TNF-α). Thus, we propose plasma levels of CXCL13 as a marker of systemic myeloid and lymphoid cell activation and inflammation in PLWH. Future studies should investigate the contribution of CXCL13 to the development of non-AIDS events and assess if plasma levels of CXCL13 can be used as a prognostic marker for the development of certain non-AIDS events including lymphoma.

T_FH_ cells in GCs and follicular DC in B cell follicles are major producers of CXCL13 owing to their expression of CXCL13 receptor CXCR5 (41, 46, 49). CXCL13 overexpression has been associated with immune activation in chronic conditions such as infection with HIV and systemic sclerosis (31, 50). Havenar-Daughton et al. studied a large longitudinal cohort of PLWH and reported a significant elevation of plasma CXCL13 with the generation of broad neutralizing antibodies (bnAbs) against HIV (41). They further correlated plasma CXCL13 levels with the magnitude of Ab responses and the frequency of T_FH_-like cells expressing ICOS in the blood of individuals' post-vaccination. Similarly, we observed an association of plasma CXCL13 with Abs such as total IgG and its subtype IgG1 in PLWH. Taken together, such findings display CXCL13 not only as another marker but also as an indicator of GC activity, which is influenced by non-specific and specific stimuli, inflammation, and vaccine response.

Our study has certain limitations that need to be considered while interpreting these findings. Causality cannot be determined as an inherent bias in observational studies like ours despite being conducted on well-defined clinical cohorts. Therefore, this study could not rule out the possibility that the dysfunction caused by chronic immune activation contributes to an increase in plasma CXCL13. Furthermore, limited sample availability did not allow us to assess whether plasma levels of CXCL13 is a marker of immune activation in known HIV reservoirs such as the gut (51). Our multivariate model did not account for potential confounding factors such as risk behaviors, economic status, smoking, alcohol, and drug abuse. Future studies should consider these limitations to further highlight the role of CXCL13 as a biomarker of immune activation among PLWH. Nevertheless, our study represents a novel and comprehensive assessment of plasma CXCL3 in the context of immune activation among PLWH with early HIV infection, before initiation of ART and while being on ART.

## Conclusion

Globally, our results suggest that elevated plasma levels of CXCL13 in PLWH are partially restored on ART and remains associated with markers of microbial translocation and systemic immune activation. Elevated CXCL13 levels can be used to monitor HIV disease progression and inflammation. Furthermore, it has potential to be used to predict the development of non-AIDS events and residual immune activation after long-term ART.

## Ethics Statement

The study was conducted in accordance with the principles of the declaration of Helsinki and all study participants provided written informed consent for study enrollment. Ethical approval was obtained from the MUHC research ethics board and by each participating medical center.

## Author Contributions

VM and RR performed the experiments, analyzed the data, wrote the first draft, and revised the final draft of the manuscript. SI, FD, IK, YZ, and MF contributed to the experiments, data analysis, and critical review of the manuscript. J-PR designed the study, contributed to data analysis, and critically reviewed the first and final draft of the manuscript. BL, CC, RT, JS, J-GB, BT, PC, RL, MD, CC-L, CT, and J-PR all contributed to recruitment and follow up of study participants and critically reviewed the manuscript. All authors have read and approved the contents of this manuscript.

### Conflict of Interest Statement

MF and YZ are employees of Associates of Cape Cod, Inc., the manufacturers of Fungitell, the (1→3)-β-D-glucan *in vitro* diagnostic kit. The remaining authors declare that the research was conducted in the absence of any commercial or financial relationships that could be construed as a potential conflict of interest.

## References

[1] Paiardini M Muller-Trutwin M. HIV-associated chronic immune activation. Immunol Rev. (2013) 254:78–101. 10.1111/imr.12079 23772616 PMC3729961

[2] Sousa AE Carneiro J Meier-Schellersheim M Grossman Z Victorino RM. CD4 T cell depletion is linked directly to immune activation in the pathogenesis of HIV-1 and HIV-2 but only indirectly to the viral load. J Immunol. (2002) 169:3400–6. 10.4049/jimmunol.169.6.3400 12218162

[3] Cao W Mehraj V Kaufmann DE Li T Routy JP. Elevation and persistence of CD8 T-cells in HIV infection: the Achilles heel in the ART era. J Int AIDS Soc. (2016) 19:20697. 10.7448/IAS.19.1.20697 26945343 PMC4779330

[4] Klatt NR Chomont N Douek DC Deeks SG. Immune activation and HIV persistence: implications for curative approaches to HIV infection. Immunol Rev. (2013) 254:326–42. 10.1111/imr.12065 23772629 PMC3694608

[5] Brenchley JM Price DA Schacker TW Asher TE Silvestri G Rao S. Microbial translocation is a cause of systemic immune activation in chronic HIV infection. Nat Med. (2006) 12:1365–71. 10.1038/nm1511 17115046

[6] Gianella S Chaillon A Mutlu EA Engen PA Voigt RM Keshavarzian A. Effect of cytomegalovirus and Epstein-Barr virus replication on intestinal mucosal gene expression and microbiome composition of HIV-infected and uninfected individuals. AIDS (2017) 31:2059–67. 10.1097/QAD.0000000000001579 28906277 PMC5654609

[7] Brites-Alves C Luz E Netto EM Ferreira T Diaz RS Pedroso C. Immune activation, proinflammatory cytokines, and conventional risks for cardiovascular disease in HIV patients: a case-control study in Bahia, Brazil. Front Immunol. (2018) 9:1469. 10.3389/fimmu.2018.01469 29997625 PMC6028567

[8] Nasi M De Biasi S Gibellini L Bianchini E Pecorini S Bacca V. Ageing and inflammation in patients with HIV infection. Clin Exp Immunol. (2017) 187:44–52. 10.1111/cei.12814 27198731 PMC5167025

[9] Klatt NR Funderburg NT Brenchley JM. Microbial translocation, immune activation, and HIV disease. Trends Microbiol. (2013) 21:6–13. 10.1016/j.tim.2012.09.001 23062765 PMC3534808

[10] Maidji E Somsouk M Rivera JM Hunt PW Stoddart CA. Replication of CMV in the gut of HIV-infected individuals and epithelial barrier dysfunction. PLoS Pathog. (2017) 13:e1006202. 10.1371/journal.ppat.1006202 28241080 PMC5328284

[11] Hensley-McBain T Berard AR Manuzak JA Miller CJ Zevin AS Polacino P. Intestinal damage precedes mucosal immune dysfunction in SIV infection. Mucosal Immunol. (2018) 11:1429–40. 10.1038/s41385-018-0032-5 29907866 PMC6162106

[12] Ancuta P Kamat A Kunstman KJ Kim EY Autissier P Wurcel A. Microbial translocation is associated with increased monocyte activation and dementia in AIDS patients. PLoS ONE (2008) 3:e2516. 10.1371/journal.pone.0002516 18575590 PMC2424175

[13] Hoenigl M Perez-Santiago J Nakazawa M de Oliveira MF Zhang Y Finkelman MA (1→3)-β-d-Glucan: a biomarker for microbial translocation in individuals with acute or early HIV infection? Front Immunol. (2016) 7:404 10.3389/fimmu.2016.00404 27752257 PMC5046804

[14] Morris A Hillenbrand M Finkelman M George MP Singh V Kessinger C. Serum (1→3)-β-D-glucan levels in HIV-infected individuals are associated with immunosuppression, inflammation, and cardiopulmonary function. J Acquir Immune Defic Syndr. (2012) 61:462–8. 10.1097/QAI.0b013e318271799b 22972021 PMC3494803

[15] Mehraj V Ramendra R Costiniuk C Lebouché B Ponte R Thomas R Circulating (1→3)-β-d-glucan as a marker of microbial translocation in HIV infection. In: The Annual Conference on Retroviruses and Opportunistic Infections CROI 2018, Poster 254. Boston, MA (2018).

[16] Hoenigl M Moser C Funderburg N Bosch R Kantor A Zhang Y. Soluble Urokinase Plasminogen Activator Receptor (suPAR) is predictive of non-AIDS events during antiretroviral therapy-mediated viral suppression. Clin Infect Dis. (2018). 10.1093/cid/ciy966 [Epub ahead of print]. 30418519 PMC6669298

[17] Siewe B Landay A Cellular and soluble immune activation markers in HIV-infected subjects. In: Hope TJ Stevenson M Richman D, editors. Encyclopedia of AIDS. New York, NY: Springer (2014). p. 1–8.

[18] Kestens L Vanham G Vereecken C Vandenbruaene M Vercauteren G Colebunders RL. Selective increase of activation antigens HLA-DR and CD38 on CD4+ CD45RO+ T lymphocytes during HIV-1 infection. Clin Exp Immunol. (1994) 95:436–41. 10.1111/j.1365-2249.1994.tb07015.x 7907956 PMC1535073

[19] Mehraj V Routy JP. Tryptophan catabolism in chronic viral infections: handling uninvited guests. Int J Tryptophan Res. (2015) 8:41–8. 10.4137/IJTR.S26862 26309411 PMC4527356

[20] Cockerham LR Siliciano JD Sinclair E O'Doherty U Palmer S Yukl SA. CD4+ and CD8+ T cell activation are associated with HIV DNA in resting CD4+ T cells. PLoS ONE (2014) 9:e110731. 10.1371/journal.pone.0110731 25340755 PMC4207702

[21] Mehraj V Jenabian MA Ponte R Lebouche B Costiniuk C Thomas R. The plasma levels of soluble ST2 as a marker of gut mucosal damage in early HIV infection. AIDS (2016) 30:1617–27. 10.1097/QAD.0000000000001105 27045377 PMC4900419

[22] Chen J Xun J Yang J Ji Y Liu L Qi T. Plasma indoleamine 2,3-dioxygenase activity is associated with the size of HIV reservoir in patients receiving antiretroviral therapy. Clin Infect Dis. (2018) ciy676. 10.1093/cid/ciy676 [Epub ahead of print]. 30107503 PMC6451994

[23] Feghali CA Wright TM. Cytokines in acute and chronic inflammation. Front Biosci. (1997) 2:d12–26. 10.2741/A171 9159205

[24] Esser R von Briesen H Brugger M Ceska M Glienke W Muller S. Secretory repertoire of HIV-infected human monocytes/macrophages. Pathobiology (1991) 59:219–22. 10.1159/000163649 1909144

[25] Scherberich JE Nockher WA. Blood monocyte phenotypes and soluble endotoxin receptor CD14 in systemic inflammatory diseases and patients with chronic renal failure. Nephrol Dial Transplant. (2000) 15:574–8. 10.1093/ndt/15.5.574 10809793

[26] Moir S Buckner CM Ho J Wang W Chen J Waldner AJ. B cells in early and chronic HIV infection: evidence for preservation of immune function associated with early initiation of antiretroviral therapy. Blood (2010) 116:5571–9. 10.1182/blood-2010-05-285528 20837780 PMC3031405

[27] Moir S Fauci AS. B cells in HIV infection and disease. Nat Rev Immunol. (2009) 9:235–45. 10.1038/nri2524 19319142 PMC2779527

[28] Velu V Mylvaganam G Ibegbu C Amara RR. Tfh1 cells in germinal centers during chronic HIV/SIV infection. Front Immunol. (2018) 9:1272. 10.3389/fimmu.2018.01272 29928280 PMC5997779

[29] Pallikkuth S de Armas L Rinaldi S Pahwa S. T follicular helper cells and B cell dysfunction in aging and HIV-1 infection. Front Immunol. (2017) 8:1380. 10.3389/fimmu.2017.01380 29109730 PMC5660291

[30] Carlsen HS Baekkevold ES Morton HC Haraldsen G Brandtzaeg P. Monocyte-like and mature macrophages produce CXCL13 (B cell-attracting chemokine 1) in inflammatory lesions with lymphoid neogenesis. Blood (2004) 104:3021–7. 10.1182/blood-2004-02-0701 15284119

[31] Widney DP Breen EC Boscardin WJ Kitchen SG Alcantar JM Smith JB. Serum levels of the homeostatic B cell chemokine, CXCL13, are elevated during HIV infection. J Interferon Cytokine Res. (2005) 25:702–6. 10.1089/jir.2005.25.702 16318584

[32] Mehraj V Cox J Lebouche B Costiniuk C Cao W Li T. Socio-economic status and time trends associated with early ART initiation following primary HIV infection in Montreal, Canada: 1996 to 2015. J Int AIDS Soc. (2018) 21:e25034. 10.1002/jia2.25034 29412520 PMC5804015

[33] Durand M Chartrand-Lefebvre C Baril JG Trottier S Trottier B Harris M. The Canadian HIV and aging cohort study - determinants of increased risk of cardio-vascular diseases in HIV-infected individuals: rationale and study protocol. BMC Infect Dis. (2017) 17:611. 10.1186/s12879-017-2692-2 28893184 PMC5594495

[34] El-Far M Kouassi P Sylla M Zhang Y Fouda A Fabre T. Proinflammatory isoforms of IL-32 as novel and robust biomarkers for control failure in HIV-infected slow progressors. Sci Rep. (2016) 6:22902. 10.1038/srep22902 26978598 PMC4792165

[35] Cao W Mehraj V Trottier B Baril JG Leblanc R Lebouche B. Early initiation rather than prolonged duration of antiretroviral therapy in HIV infection contributes to the normalization of CD8 T-cell counts. Clin Infect Dis. (2016) 62:250–7. 10.1093/cid/civ809 26349551 PMC4690481

[36] Jenabian MA El-Far M Vyboh K Kema I Costiniuk CT Thomas R. Immunosuppressive tryptophan catabolism and gut mucosal dysfunction following early HIV infection. J Infect Dis. (2015) 212:355–66. 10.1093/infdis/jiv037 25616404

[37] Jenabian MA Patel M Kema I Kanagaratham C Radzioch D Thebault P. Distinct tryptophan catabolism and Th17/Treg balance in HIV progressors and elite controllers. PLoS ONE (2013) 8:e78146. 10.1371/journal.pone.0078146 24147117 PMC3797729

[38] Zaunders J van Bockel D. Innate and adaptive immunity in long-term non-progression in HIV disease. Front Immunol. (2013) 4:95. 10.3389/fimmu.2013.00095 23630526 PMC3633949

[39] Lurain NS Hanson BA Hotton AL Weber KM Cohen MH Landay AL. The association of human cytomegalovirus with biomarkers of inflammation and immune activation in HIV-1-infected women. AIDS Res Hum Retroviruses (2016) 32:134–43. 10.1089/aid.2015.0169 26422187 PMC4761818

[40] Chen J Ramendra R Lu H Routy JP The early bird gets the worm: benefits and future directions with early antiretroviral therapy initiation in primary HIV infection. Fut. Virol. (2018) 13, 779–786. 10.2217/fvl-2018-0110

[41] Havenar-Daughton C Lindqvist M Heit A Wu JE Reiss SM Kendric K. CXCL13 is a plasma biomarker of germinal center activity. Proc Natl Acad Sci USA. (2016) 113:2702–7. 10.1073/pnas.1520112113 26908875 PMC4790995

[42] Camilli G Tabouret G Quintin J. The complexity of fungal beta-Glucan in health and disease: effects on the mononuclear phagocyte system. Front Immunol. (2018) 9:673. 10.3389/fimmu.2018.00673 29755450 PMC5932370

[43] Farhour Z Mehraj V Chen J Ramendra R Lu H Routy JP. Use of (1→3)-β-d-glucan for diagnosis and management of invasive mycoses in HIV-infected patients. Mycoses (2018) 61:718–22. 10.1111/myc.12797 29855088 PMC6175469

[44] Lu W Mehraj V Vyboh K Cao W Li T Routy JP. CD4:CD8 ratio as a frontier marker for clinical outcome, immune dysfunction and viral reservoir size in virologically suppressed HIV-positive patients. J Int AIDS Soc. (2015) 18:20052. 10.7448/IAS.18.1.20052 26130226 PMC4486418

[45] Vidya Vijayan KK Karthigeyan KP Tripathi SP Hanna LE. Pathophysiology of CD4+ T-cell depletion in HIV-1 and HIV-2 infections. Front Immunol. (2017) 8:580. 10.3389/fimmu.2017.00580 28588579 PMC5440548

[46] Rasheed AU Rahn HP Sallusto F Lipp M Muller G. Follicular B helper T cell activity is confined to CXCR5(hi)ICOS(hi) CD4 T cells and is independent of CD57 expression. Eur J Immunol. (2006) 36:1892–903. 10.1002/eji.200636136 16791882

[47] Gu-Trantien C Migliori E Buisseret L de Wind A Brohee S Garaud S. CXCL13-producing TFH cells link immune suppression and adaptive memory in human breast cancer. JCI Insight. (2017) 2:91487. 10.1172/jci.insight.91487 28570278 PMC5453706

[48] Cohen KW Dugast AS Alter G McElrath MJ Stamatatos L. HIV-1 single-stranded RNA induces CXCL13 secretion in human monocytes via TLR7 activation and plasmacytoid dendritic cell-derived type I IFN. J Immunol. (2015) 194:2769–75. 10.4049/jimmunol.1400952 25667414 PMC4363079

[49] Wang X Cho B Suzuki K Xu Y Green JA An J. Follicular dendritic cells help establish follicle identity and promote B cell retention in germinal centers. J Exp Med. (2011) 208:2497–510. 10.1084/jem.20111449 22042977 PMC3256970

[50] Taniguchi T Miyagawa T Toyama S Yamashita T Nakamura K Saigusa R. CXCL13 produced by macrophages due to Fli1 deficiency may contribute to the development of tissue fibrosis, vasculopathy and immune activation in systemic sclerosis. Exp Dermatol. (2018) 27:1030–7. 10.1111/exd.13724 29947047

[51] Mehraj V Ghali P Ramendra R Costiniuk C Lebouche B Ponte R. The evaluation of risk-benefit ratio for gut tissue sampling in HIV cure research. J Virus Erad. (2017) 3:212–7. 29057085 10.1016/S2055-6640(20)30316-2 PMC5632548

